# Electrodialytic Transport Properties of Anion-Exchange Membranes Prepared from Poly(vinyl alcohol) and Poly(vinyl alcohol-*co*-methacryloyl aminopropyl trimethyl ammonium chloride)

**DOI:** 10.3390/membranes3010001

**Published:** 2012-12-31

**Authors:** Atsushi Jikihara, Reina Ohashi, Yuriko Kakihana, Mitsuru Higa, Kenichi Kobayashi

**Affiliations:** 1 Graduate School of Science and Engineering, Yamaguchi University, 2-16-1 Tokiwadai, Ube, Yamaguchi 755-8611, Japan; E-Mails: atsushi_jikihara@kuraray.co.jp (A.J.); j008fc@yahoo.co.jp (R.O.); kakihana@yamaguchi-u.ac.jp (Y.K.); 2 VA-related Polymers Research Laboratory, Kurashiki Research Center, Kuraray, 2045-1 Sakazu, Kurashiki, Okayama 710-0801, Japan; E-Mail: kenichi_kobayashi@kuraray.co.jp

**Keywords:** anion-exchange membrane, poly(vinyl alcohol-*co*-methacryloyl aminopropyl trimethyl ammonium chloride), poly(vinyl alcohol), water content, charge density, membrane resistance

## Abstract

Random-type anion-exchange membranes (AEMs) have been prepared by blending poly(vinyl alcohol) (PVA) and the random copolymer-type polycation, poly(vinyl alcohol-*co*-methacryloyl aminopropyl trimethyl ammonium chloride) at various molar percentages of anion-exchange groups to vinyl alcohol groups, *C*_pc_, and by cross-linking the PVA chains with glutaraldehyde (GA) solution at various GA concentrations, *C*_GA_. The characteristics of the random-type AEMs were compared with blend-type AEMs prepared in our previous study. At equal molar percentages of the anion exchange groups, the water content of the random-type AEMs was lower than that of the blend-type AEMs. The effective charge density of the random-type AEMs increased with increasing *C*_pc_ and reached a maximum value. Further, the maximum value of the effective charge density increased with increasing *C*_GA_. The maximum value of the effective charge density, 0.42 mol/dm^3^, was obtained for the random-type AEM with *C*_pc_ = 4.2 mol % and *C*_GA_ = 0.15 vol %. A comparison of the random-type and blend-type AEMs with almost the same *C*_pc_ showed that the random-type AEMs had lower membrane resistance than the blend-type ones. The membrane resistance and dynamic transport number of the random-type AEM with *C*_pc_ = 6.0 mol % and *C*_GA_ = 0.15 vol % were 4.8 Ω cm^2^ and 0.83, respectively.

## 1. Introduction

Anion exchange membranes (AEMs) have been applied in various industrial fields [1,2]: The separation of environmental polluting metal ions from hard water [3], alkaline direct methanol cells [4], the electrodialytic concentration or desalination of electrolyte solutions [5], *etc.* At present, almost all of the commercially available AEMs for electrodialysis have a styrene-*co*-divinylbenzene based matrix. The drawbacks of these membranes are the difficulty of controlling the membrane structure because the copolymerization and cross-linking processes undergo simultaneously and their high manufacturing cost. Recently, many studies on the preparation of novel ion exchange membranes (IEMs) have been reported to overcome these problems [6,7]. IEMs can be prepared by mixing water-soluble base polymers and a polyelectrolyte and then cross-linking the base polymer. The membrane thus obtained has a semi-interpenetrating network (semi-IPN) structure in which polyelectrolyte chains are immobilized in a polymer cross-linked network matrix. The ion exchange capacity of the membranes can be controlled easily by changing the polymer ratio of water-swollen base polymer to polyelectrolyte. Poly(vinyl alcohol) (PVA) is one of the most popular water-soluble polymers. PVA is a polyhydroxy polymer that has been studied intensively as water-soluble base polymer because of its good film forming and physical properties, high hydrophilicity, processability, biocompatibility, and good chemical resistance. Cation-exchange membranes with a semi-IPN structure have been prepared by blending PVA and a polyanion such as poly(styrene sulfonic acid) [8], poly(acrylic acid) [9], poly(styrene sulfonic acid-*co*-maleic acid) [10], poly(vinyl alcohol-*co*-2-acrylamido-2-methylpropane sulfonic acid) [11,12], and by using PVA and sulfosuccinic acid [13], *etc.* Anion-exchange membranes prepared by blending PVA and a polycation have also been investigated [14,15,16]. AEMs with a semi-IPN structure have been prepared by blending PVA and a polycation such as poly(acrylamide-*co*-diallyldimethylammonium chloride) [17], poly(allyl amine) [18], *etc.* However those electrodialytic transport properties were not enough to compete with commercially available AEMs. An additional disadvantage of AEMs with semi-IPN structure is the low long-term stability when immersed in solution due to the dissolution of uncross-linked water-soluble polyelectrolytes in the network into the solution.

**Figure 1:**
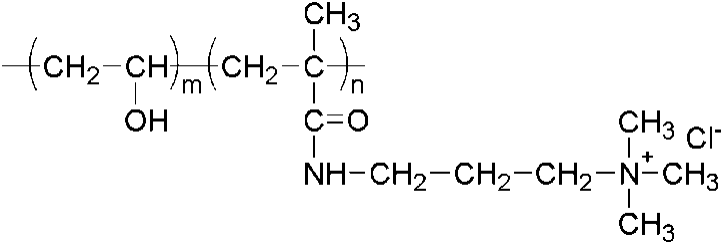
Chemical structure of poly(vinyl alcohol-*co*-methacryloyl aminopropyl trimethyl ammonium chloride).

The aim of this study is to show advantages of using random copolymer-type PVA, poly(vinyl alcohol-*co*-methacryloyl aminopropyl trimethyl ammonium chloride: MAPTAC), as a base material for AEM instead of using a blend-type one as in our previous study [18]. The chemical structure of PVA-*co*-MAPTAC is shown in Figure 1. We have investigated the differences of the following ionic transport properties for electrodialysis applications: effective charge density, membrane resistance, and transport number of ions, between PVA-based AEMs prepared by the two different methods.

## 2. Results and Discussion

### 2.1. Water Content as a Function of *C*_pc_

**Figure 2:**
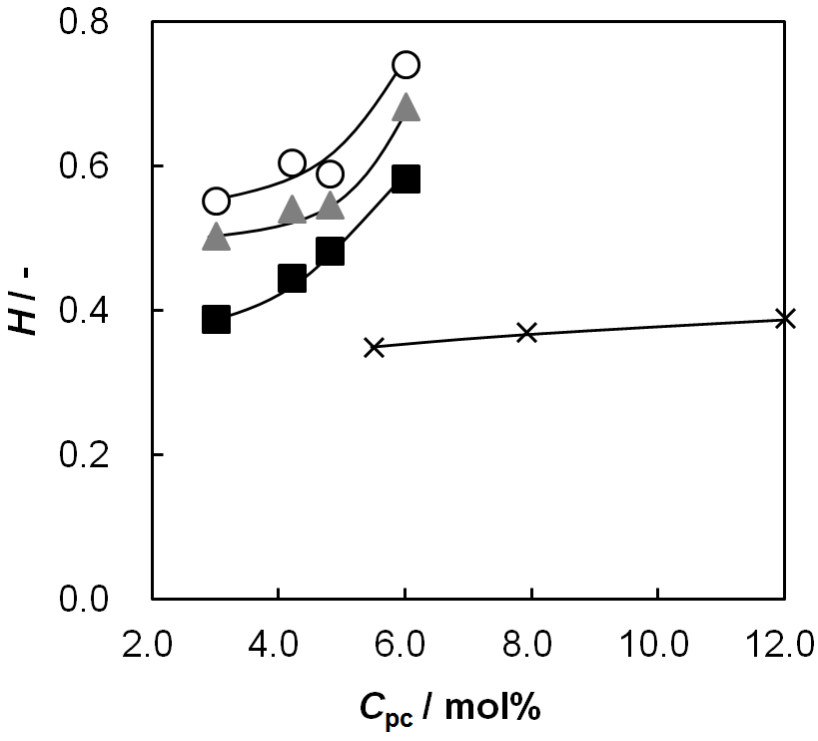
Water content, *H*, of anion-exchange membranes (AEMs) as a function of molar percentage of anion-exchange groups to vinyl alcohol groups, *C*_pc_. Open and closed symbols are the data of the random copolymer AEMs where the glutaraldehyde concentrations (*C*_GA_) are: Circles, 0.01 vol %; triangles, 0.05 vol %; squares, 0.15 vol %. Cross marks are the data of polymer blend AEMs as a control where *C*_GA_ is 0.075 vol %.

The water content of an IEM is an important property because both the effective charge density and electric resistance of the membrane depend on the water content. Figure 2 shows the water content of the AEMs prepared in this study as a function of *C*_pc_. Here *C*_pc_ is identified as a molar percentage of cation-exchange groups in the membrane. Data obtained for the polymer blend-type AEMs quoted from our previous study [18] are also shown in order to compare the characteristics of them. The water content increased with increasing *C*_pc_, because the osmotic pressure in the membranes increased with an increase in the number of charged groups in the membranes. Further, the water content decreased with increasing glutaraldehyde (GA) concentration, *C*_GA_, because of the increase in the number of chemical cross-linking points with increasing *C*_GA_. A comparison of the water content of the random-type AEMs with that of the polymer blend-type AEMs at the same *C*_pc_ shows that the water content of the polymer blend-type AEMs is lower than that of the random-type. For example, in AEMs with *C*_pc_ = 5.4 mol %, the polymer blend-type AEMs showed smaller water content than the random-type AEMs although *C*_GA_ of the former was higher than that of the latter. This difference is attributed to the difference in the morphology among AEMs. The random-type AEM has a uniform morphology because the ion-exchange groups randomly connect to PVA chains. On the other hand, the blend-type AEM has a phase-separated morphology at large sizes because it consists of two different kinds of polymers, PVA and poly(allyl amine). Hence some parts of the poly(allyl amine) chains dissolve into the cross-linking solution during the cross-linking process. Therefore, the water content of the blend-type AEMs is almost independent of the *C*_pc_ content as shown in Figure 2.

### 2.2. IEC of the AEMs

Table 1 shows the molar percentage of the anionic-exchange groups, *C*_pc_, and *IEC* of the AEMs (RPA-*X*) along with the values for a commercially available AEM, Neosepta AMX (ASTOM Corp.: Tokyo, Japan). The experimental *IEC* values are about 30%–40% of the calculations. This might be attributed to the fact that the same part of the charged groups in the random-copolymer were enclosed with the crystal regions in the random-type AEMs, and that the counter-ion of the enclosed charged groups may not be able to exchange ions with that of the external solution. The *IEC* of the commercial AMX is 1.4 meq/g dry AEM. Hence, the *IEC* of RPA-4 is about one third of that of AMX.

### 2.3. The Effective Charge Density of the Membranes as a Function of *C*_pc_

**Figure 3:**
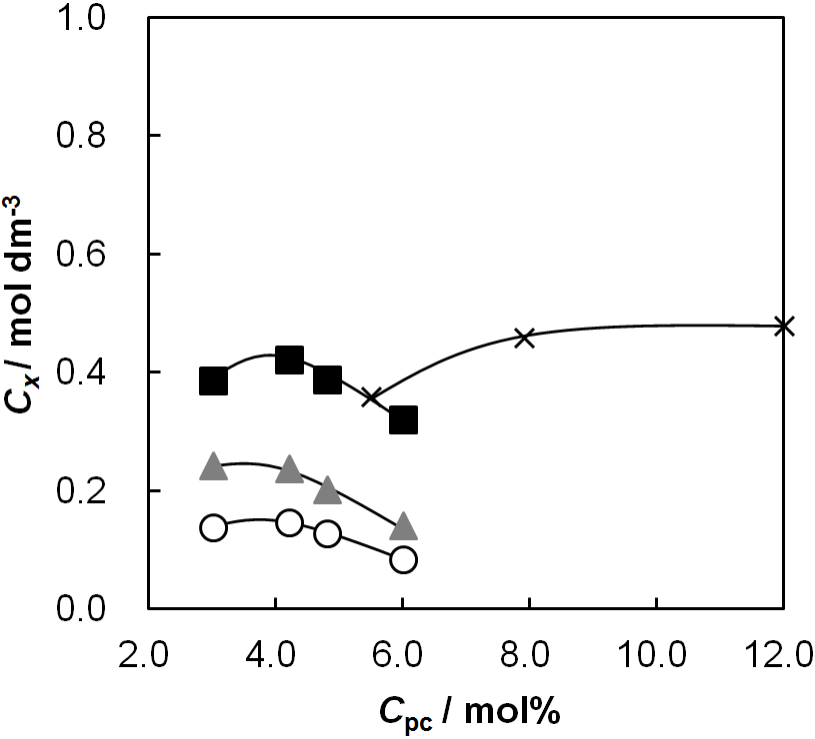
Effective charge density, *C_x_*, of the AEMs as a function of the molar percentage of the anion-exchange groups, *C*_pc_. The definition of symbols in this figure is the same as in Figure 2.

The permselectivity of an IEM is best determined via membrane potential measurements as effective charge density, because the higher the effective charge density of a membrane, the higher the counter ion permselectivity. Figure 3 shows the effective charge density of the membranes as a function of *C*_pc_. The effective charge density increased with *C*_pc_ and reached a maximum value. The effective charge density is proportional to the charge density, which is defined as the division of *IEC* by the water content, *H*:

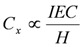
(1)

**Table 1:** The molar percentage of anion-exchange groups, *C*_pc_, and ion-exchange capacity, *IEC*, of the base membrane: The AEMs in this study were prepared from the base membranes: molar percentage of the anionic-exchange groups, *C*_pc_, and *IEC* of the AEMs (RPA-*X*) (*X* = 1–4), changing GA concentrations.

Sample	*C*_pc_ (mol %)	*IEC* (meq/g dry-CEM)
RPA-1	3.0	0.22 (0.60 ^a^)
RPA-2	4.2	0.27 (0.81)
RPA-3	4.8	0.30 (0.90)
RPA-4	6.0	0.39 (1.09)
AMX ^a^	–	1.4

^a^ A commercial AEM (Neosepta AMX, Astom Corp.: Tokyo, Japan).

*IEC* was almost proportional to *C*_pc_ as shown in Table 1. The water content also increased with *C*_pc_ as shown in Figure 2. Hence, in the first stage of the effective charge density-*C*_pc_ curves, the effective charge density increases with increasing *C*_pc_ because of the increase in *IEC*. At high *C*_pc_, the effect of the increase of water content on the effective charge density is larger than that of the increase in *IEC*. As a result, the effective charge density decreased after reaching a maximum value. The maximum value of the effective charge density increases with increasing *C*_GA_ because the water content decreases with increasing *C*_GA_. The maximum value of the effective charge density was 0.42 mol/dm^3^ for the random-type AEM having *C*_pc_ = 4.2 mol % and *C*_GA_ = 0.15 vol %. The effective charge density of AMX measured under the same conditions is 1.6 mol/dm^3^. Thus, the effective charge density of the random-type AEM was almost one fourth of that of the commercially available AEM.

### 2.4. Membrane Resistance as a Function of *C*_pc_

**Figure 4:**
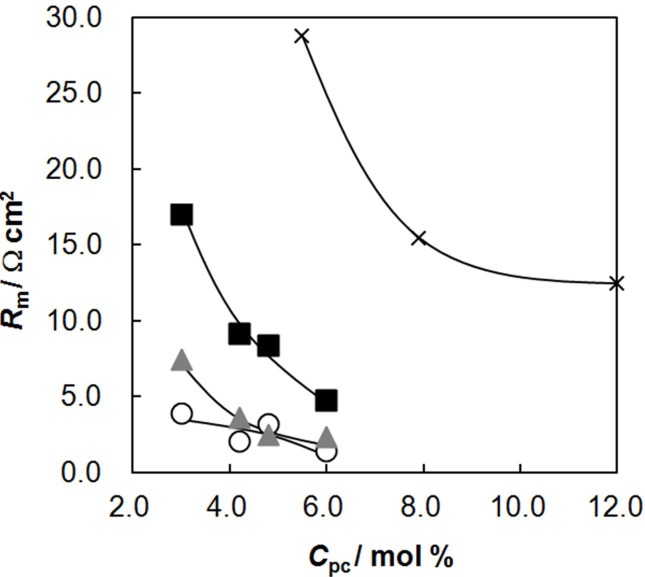
Membrane resistance, *R*_m_, of the AEMs as a function of the molar percentage of the anion-exchange groups, *C*_pc_. The definition of symbols in this figure is the same as in Figure 2.

The membrane resistance is important for energy-saving electrodialysis. Figure 4 shows the membrane resistance as a function of *C*_pc_. The membrane resistance decreased with increasing *C*_pc_ because of the increase in the water content and *IEC* with increasing *C*_pc_. The resistance increased with increasing *C*_GA_ because of the decrease in the water content. The lowest membrane resistance of 1.4 Ω cm^2^ was obtained for the random-type AEMs at *C*_pc_ = 6.0 mol % and *C*_GA_ = 0.01 vol %. A comparison of the membrane resistance of the random-type AEMs with that of the polymer blend-type ones at the same molar percentage of *C*_pc_ showed that the membrane resistance of the random-type AEMs was lower than that of the blend-type ones. For example, in AEMs with *C*_pc_ = 6.0 mol %, the membrane resistance of the blend-type AEMs was far larger than that of the random-type AEMs although *C*_GA_ of the random-type AEM was higher than that of the blend-type one. This difference also indicates that the distribution of the ion-exchange parts is different among AEMs. In the case of polymer-blend AEMs some portion of the polycation chains located on the membrane surfaces will be dissolved in solution and leaked out from the membrane. This will cause high electrical resistance near the membrane surfaces.

### 2.5. Membrane Resistance as a Function of Water Content

The membrane resistance depends on the mobility and concentration of ions in an IEM. It is difficult to measure experimentally the ionic mobility in a water-swollen membrane. To estimate the ionic mobility, many authors have replaced the mobility as a function of both the mobility in an aqueous solution, *w*_i_ and factors such as membrane geometry. Mackie and Meares [19] stochastically dealt with these factors as a function of the water content of a swollen-membrane. Consequently the dependence of membrane resistance on water is given as the following equation:

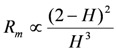
(2)

**Figure 5:**
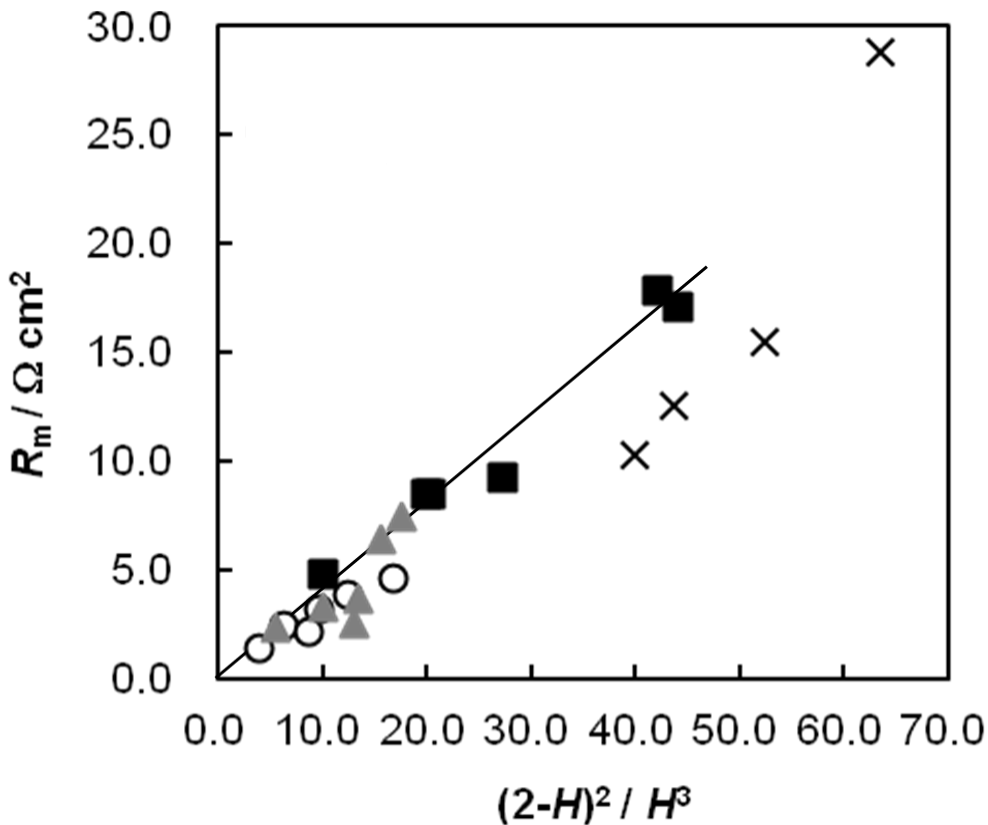
Membrane resistance, *R*_m_, of the AEMs as a function of water content, (2 − *H*)^2^/*H*^3^. The definition of symbols in this figure is the same as in Figure 2.

Figure 5 shows the membrane resistance as a function of water content, (2 − *H*)^2^/*H*^3^, where the resistances of all the random-type AEMs are almost linear. This means that the resistance of the random-type AEMs can be estimated from the value of the water content.

### 2.6. Dynamic State Transport Number of the AEMs as a Function of *C*_pc_

**Figure 6:**
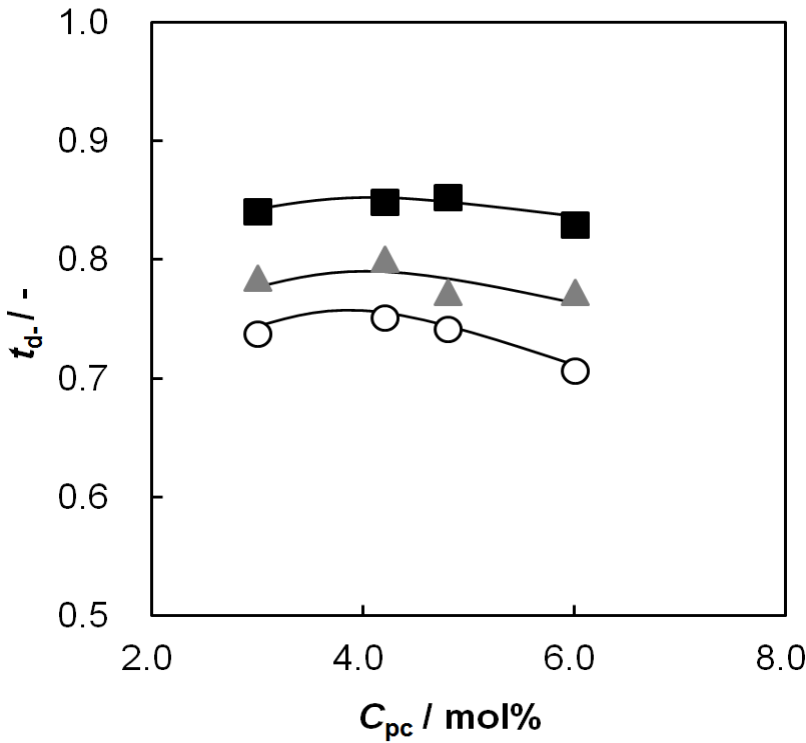
Dynamic state transport number, *t*_d−_, of the AEMs as a function of the molar percentage of the anion-exchange groups, *C*_pc_. The definition of symbols in this figure is the same as in Figure 2.

The dynamic state transport number relates to the counter-ion permselectivity of an IEM in electrodialysis. An IEM with a transport number of 1.0 can allow the permeation of only the counter ions in an electrodialysis system. Figure 6 shows the dynamic state transport number of the AEMs as a function of *C*_pc_. The transport number of the random-type AEMs increased with increasing *C*_pc_, and then decreased after reaching a maximum value at *C*_pc_ ≈ 4.2 mol %. As mentioned above, this trend is attributed to the fact that the effective charge density has a maximum value at *C*_pc_ = 4.2 mol %. The transport number increased with increasing *C*_GA_. These results indicate that the transport number of the membranes can be controlled by changing the cross-linking conditions. The dynamic transport number of the random-type AEM with *C*_pc_ = 4.2 mol % and *C*_GA_ = 0.15 vol % is 0.85, while that of AMX is 0.98 under the same conditions (0.5 mol/dm^3^ NaCl), indicating that the random-type AEM applies to desalination under lower salt concentration than 0.5 mol/dm^3^ NaCl.

### 2.7. Relationship between Membrane Resistance and Dynamic State Transport Number

**Figure 7:**
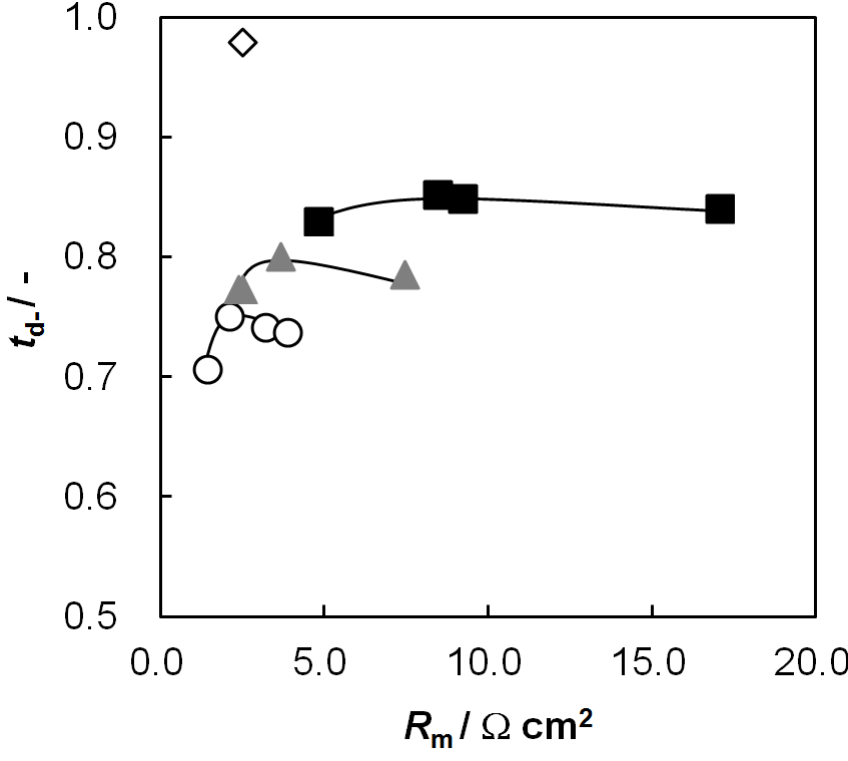
Dynamic state transport number, *t*_d−_, of AEMs as a function of membrane resistance, *R*_m_. The definition of symbols in this figure is the same as in Figure 2. Open diamond represents the data of the commercially-available AEM, Neosepta AMX (ASTOM Corp.: Tokyo, Japan).

An ion exchange membrane having both high ion permselectivity and low membrane resistance is desirable in electrodialysis processes. Figure 7 shows the relationship between the membrane resistance and dynamic state transport number of the membranes. The membrane placed on the upper left-hand side of the figure has a high performance for electrodialysis processes. The membrane resistance decreases and the dynamic state transport number increases with increasing *C*_pc_. The transport number of the membrane increases with increasing *C*_GA_. The dynamic transport number and membrane resistance of the random-type AEM at *C*_pc_ = 6.0 mol % and *C*_GA_ = 0.15 vol % were 0.83 and 4.8 Ω cm^2^, respectively, while those of AMX were 0.98 and 2.5 Ω cm^2^, respectively. This means that the random-type AEMs have lower ionic selectivity than AMX at high concentration of saline solutions.

### 2.8. Relationship between Dynamic State Transport Number and Effective Charge Density

**Figure 8:**
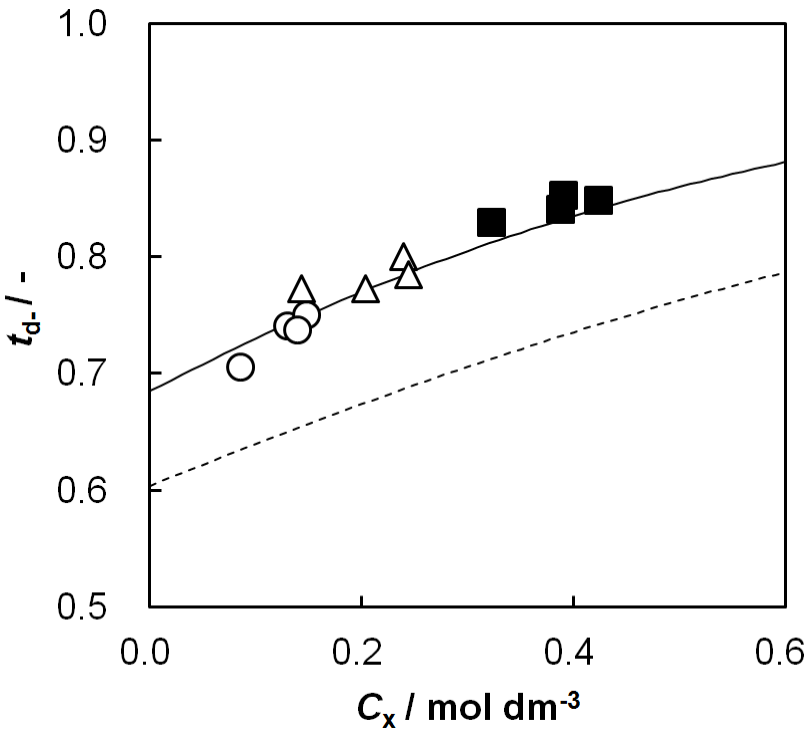
Dynamic state transport number, *t*_d−_, of AEMs as a function of the effective charge density, *C_x_*. The definition of symbols in this figure is the same as in Figure 2. The calculations: broken curve, *β* = 0.66; solid curve, *β* = 0.46.

Figure 8 shows the dynamic state transport number as a function of the effective charge density. The transport number increased with increasing effective charge density and was almost on a curve independent of the other membrane parameters: *IEC* and *C*_GA_. 

The Donnan theory on an IEM gives the relationship between the transport number and effective charge density as [20]:

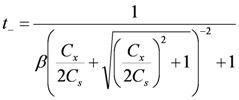
(3)
where *C*_s_ is the NaCl concentration at the transport number measurement (0.5 mol/dm^3^).

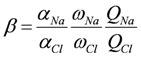
(4)
where *α_i_* is the correction factor of the i-th ion in the Mackie and Meares theory; *w_Na_* and *w_Cl_* are the mobilities of Na^+^ and Cl^−^ ions in the membrane, and *Q_i_* is the factor related to the standard chemical potential difference and the activity coefficient as

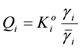
(5)

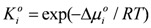
(6)
where Δ*μ*0 i is the standard chemical potential difference of the ion, respectively, between the membrane and an aqueous solution; 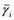 and *γ*_i_ are the corresponding activity coefficients in the membrane and the solution. The values of the mobility of Na^+^ and Cl^−^ ions in an aqueous solution are 5.4 × 10^−13^ mol m^2^ J^−1^ s^−1 ^and 7.9 × 10^−13^ mol m^2^ J^−1^ s^−1^, respectively [21]. Hence, the ratio of *w_Na_* to *w_Cl_* is 0.66. In the case of assuming that *α_Na_*/*α_Cl_* = 1 and *Q_Na_*/*Q_Cl_* = 1, the calculation of *β* = 0.66 is lower than the experiments as shown in the broken curve in Figure 7. The calculation of *β* = 0.46 as shown in the solid curve is in good agreement with the experiments. This means that the ratio of the correction factor, *α_Na_*/*α_Cl_*, and/or the ratio, *Q_Na_*/*Q_Cl_*, is less than 1.0. The correction factor of the highly hydrated ion Ca^2+^ in PVA-based membranes is smaller than that of the Cl^−^ (*α_Ca_* = 0.60, *α_Cl_* = 0.94) [21]. The radius of the hydrated Na^+ ^is lower than that of the Cl^−^. Hence, *α_Na_*/*α_Cl_* is lower than 1.0. The result indicates that the transport number can be estimated from the value of the effective charge density.

## 3. Experimental Section

### 3.1. Materials

PVA117 (degree of hydrolysis: 98.5 mol %, viscosity-average degree of polymerization: 1700) and poly(vinyl alcohol-*co-*methacryloyl aminopropyl trimethyl ammonium chloride: MAPTAC) (degree of MAPTAC modification: 4 mol %, degree of hydrolysis: 98.5 mol %, viscosity-average degree of polymerization: 1700) were obtained from Kuraray Co., Ltd.: Tokyo, Japan. Glutaraldehyde (GA) (25 wt % solution in water) was of analytical grade and was obtained from Wako Pure Chemical Industries. Sulfuric acid and sodium sulfate were of analytical grade and were obtained from Nakarai Tesque.

### 3.2. Preparation of Random-Type AEMs

**Figure 9:**
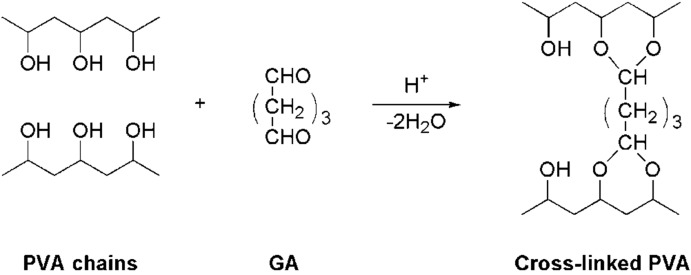
Scheme of chemical cross-linking of poly(vinyl alcohol) (PVA) using glutaraldehyde (GA).

Self-standing base membranes, BM-*X* (*X* = 1–4) for random-type AEMs were prepared by casting an aqueous solution of a mixture of PVA and PVA-*co*-MAPTAC on a plastic plate and then drying the mixture over a hot stage (NISSIN, NH-45N) overnight at 50 °C. The thickness of the base membranes was *ca.* 0.1 mm. The weight percentage of PVA-*co*-MAPTAC to PVA in the mixture solution was varied to control the molar percentage of anion-exchange groups to vinyl alcohol groups (*C*_pc_) in BM-*X*, as shown in Table 1. The random-type AEMs, RPA-*X*, were prepared by annealing BM-*X* at 160 °C for 30 min under vacuum to induce physical cross-linking between the PVA chains because PVA is a semi-crystalline polymer and the crystalline region acts as a physical cross-link point [22]. Chemical cross-linking was induced by immersing the membranes in an aqueous solution of various concentrations of GA, 0.05 mol/dm^3^ of H_2_SO_4_ (pH = 1), and 2.0 mol/dm^3^ of Na_2_SO_4_ at 25 °C for 24 h to control the water content of the membranes in the equilibrium swelling state in deionized water. Figure 9 shows the chemical cross-linking mechanism of PVA and GA.

### 3.3. Measurement of Membrane Water Content

The water content, *H*, of the membranes was measured as follows: A base membrane was weighed in the dry state after annealing and chemical cross-linking. The membrane was immersed in deionized water at 25 °C for 7 days. The membranes were removed from water, dabbed with a filter paper to remove excess water on the membrane surfaces, and weighed in the wet state. For the sake of simplicity, we assume the volume between the water phase and the polymer phase of a swollen membrane to be additive. The volumetric water content, *H*, is calculated from the weights in the wet state, *W*_w_, and in the dry state, *W*_d_, as shown below:

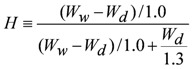
(7)
where 1.0 and 1.3 are the densities of water and PVA [23], respectively.

### 3.4. Measurement of Ion-Exchange Capacity (IEC)

The ion-exchange capacity (*IEC*) of an AEM is an important parameter because the ionic transport properties of the AEM depend on the amount and species of the ion exchange groups. *IEC* is expressed as milliequivalent per gram of membrane (meq/g dry AEM) and was determined as follows: A sample membrane was immersed in 0.10 mol/dm^3^ KCl solution for 3 h before measuring *IEC*. The membrane was rinsed with deionized water to remove non-exchange KCl electrolyte adsorbed on the membranes and was then immersed in 0.20 mol/dm^3^ of NaNO_3_ with a volume of 50 cm^3^ under stirring for 12 h to achieve the complete exchange of Cl^−^ ions in the membrane with NO_3_^−^ ions in the solution. The concentration of Cl^−^ ions in the solution, *C*_Cl _was determined by using an ion chromatograph (Dionex ICS-1500). The membrane was dried under vacuum for 24 h and was weighed in the dry state, *W_d_*. *IEC* of the base membrane was calculated using the following equation:

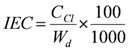
(8)

### 3.5. Determination of Membrane Effective Charge Density

Membrane effective charge density is one of the most important factors for estimating the permselectivity between ions with different charge and valence in an ion exchange membrane. In order to estimate the effective charge density, the membrane potentials, Δ*ϕ*, were measured as a function of the KCl concentration in the two chambers of the apparatus described elsewhere [18]. One chamber of the cell was filled with KCl solution of various concentrations, *C*_o_, while the other chamber was filled with KCl solutions with concentration, *C*_d_, which is five times higher than that in the former (*r* = *C*_d_/*C*_o_= 5). The membrane potential between the solutions in the two chambers was measured at 25.0 ± 0.5 °C using Ag/AgCl electrodes (TOA HS- 205C) with a salt bridge (3.0 mol/dm^3^ KCl) and a voltmeter HM-20E (TOA Corp.: Tokyo, Japan). The membrane effective charge density, *C_x_*, was calculated from the measured membrane potential using the following equation [24,25]:

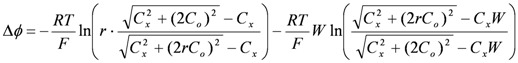
(9)
where *W* ≡ (*w_k _- w_Cl _*) / (*w_k _+ w_Cl _*); *w_k_* and *w_Cl_* are the K^+^ and Cl^−^ mobilities in a membrane, respectively; *F*, *R* and *T* are the Faraday constant, gas constant and the absolute temperature, respectively. Parameters *W* and *C_x_* are adjusted so that the left-hand side of Equation (9) fits the experimental data at various KCl concentrations.

### 3.6. Measurement of Membrane Resistance

The electrical resistance of a sample membrane was measured by using a handmade acrylic plastic cell composed of two parts separated by the membrane, as described elsewhere [18], with an LCR meter AD-5827 (A&D Corp. Ltd.: Tokyo, Japan) operated at 10 KHz AC. An amount of 0.5 mol/dm^3^ of NaCl solution was poured into the two cell compartments, after which the electrical resistance, *R*_o_, was measured at 25.0 ± 0.5 °C in a water bath. Subsequently, a sample membrane was set in the cell and the resistance, *R*_s_, was measured again under the same conditions. The difference between *R*_s_ and *R*_o_ gives the membrane resistance, *R*_m_.

### 3.7. Measurement of Dynamic State Transport Number

**Figure 10:**
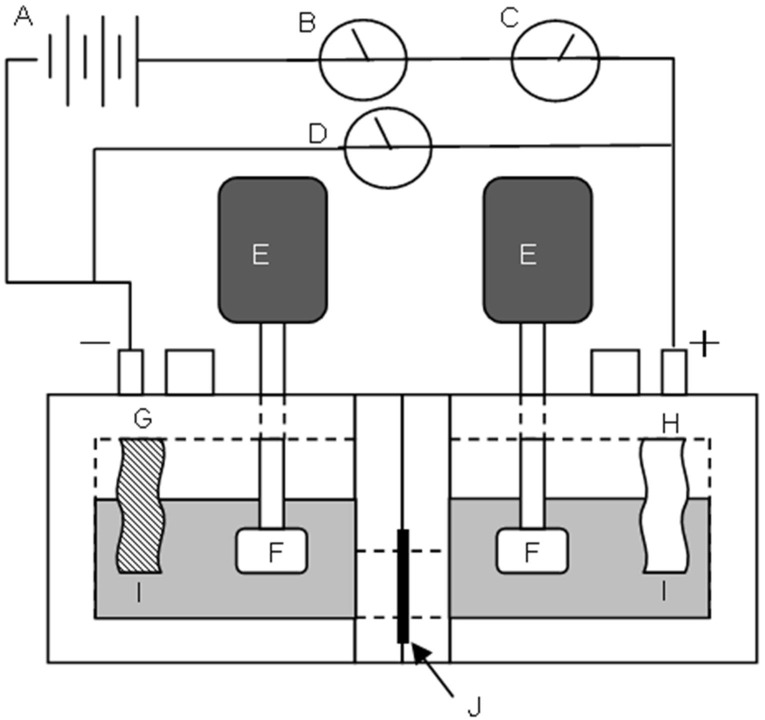
Apparatus for measuring the dynamic state transport number. A: power supply; B: ampere meter; C: coulomb meter; D: voltmeter; E: motor; F: stirrer; G: cathode electrode; H: anode electrode; I: 0.5 mol/dm^3^ NaCl solution; J: sample membrane. The effective area of the cell is 8.0 cm^2^.

The dynamic state transport number, *t*_d−_, of a membrane was determined by electrodialysis carried out using the handmade acrylic plastic cell with two parts separated by the membrane as shown in Figure 10. 

The amount of ions transported through the membrane during electrodialysis was measured using a conductivity meter (HORIBA 3552-10D). The dynamic state transport number was obtained using the following equation:

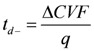
(10)
where ∆*C*, *V* and *q* are the concentration change of the transported ions, the volume of the measurement solution, and the amount of electricity passing through the membrane during the electrodialysis, respectively.

## 4. Conclusions

In this study, PVA-based AEMs were prepared by blending PVA with the random copolymer-type PVA-based polycation. The water content of the random-type AEMs was higher than that of the polymer blend-type AEMs when the molar percentage of the cation-exchange groups, *C*_pc_, was almost the same for both the AEMs. The effective charge density of the random-type AEMs increased with an increase in *C*_pc_ and reached a maximum value. The maximum value increased with increasing crosslinker concentration, *C*_GA_, and the value of 0.42 mol/dm^3^ was obtained with *C*_pa_ = 4.2 mol % and *C*_GA_ = 0.15 vol %, which was almost one fourth of the value of the commercially available AEM, AMX. A comparison of the random-type and polymer blend-type AEMs having almost the same membrane resistance showed that the random-type AEMs had lower membrane resistance than polymer blend-type ones. The membrane resistance of random-type AEMs can be reduced by optimizing the molar percentage of the anion-exchange groups to vinyl alcohol groups and the cross-linking conditions. The transport number and membrane resistance of the random-type AEM with *C*_pc_ = 6.0 mol % and *C*_GA_ = 0.15 vol % were 0.83 and 4.8 Ω cm^2^, respectively, while those of AMX were 0.98 and 2.5 Ω cm^2^, respectively.

The random-type AEMs should have an advantage of production cost compared to the commercially available ones. Hence, the AEMs will be potentially applicable to electrodialytic desalination at low salt concentration like brackish water, food and medical products. The next subject of this study is to examine the desalination performance of the random-type AEMs with a lab-scale electrodialyzer.

## Nomenclatures

*C*: ionic concentration in a solution
*C*_pc_: polycation content in the membrane
*C*_GA_: glutaraldehyde concentration
*C_x_*: the charge density in an anion exchange membrane
*F*: Faraday’s constant
*H*: water content
*IEC*: Ion exchange capacity
*q*: the amount of electricity passing through a membrane during electrodialylsis
*r*: KCl concentration ratio between the high- and low-concentration chambers
*R*: gas constant
*R*_o_: measured resistance without a sample membrane
*R*_s_: measured resistance with a sample membrane
*R*_m_: membrane resistance: *R*_m_* = R*_s_*− R*_o_
*t*: transport number
*T*: absolute temperature
*W*_d_: weight in dry state
*W*_w_: weight in wet state

## Greek Symbols

*α*: correction factor
activity coefficients in the membrane
*γ*: activity coefficients in the solution
Δ*ϕ*: membrane potential
Δ*μ*^0^: standard chemical potential
*w*: ionic mobility

## Subscripts

*Cl*: chloride ion
*D*: high-concentration chamber
*K*: potassium ion
*Na*: sodium ion
*s*: electrolyte solution
*o*: low-concentration chamber
